# Formation, Growth, and Shrinkage of Voids in Lithium Metal in Contact With an LLZO Electrolyte

**DOI:** 10.1021/acsami.5c09594

**Published:** 2025-10-05

**Authors:** Sabrina Lang, Lukas Hennerici, Dominik Kramer, Diana Avadanii, Stefan Mück, Mario Linz, Jaroslaw Kita, Ralf Moos, Reiner Mönig

**Affiliations:** † Institute for Applied Materials, 537456 Karlsruhe Institute of Technology, 76344 Eggenstein-Leopoldshafen, Germany; ‡ Department for Functional Materials, 26523 University of Bayreuth, 95447 Bayreuth, Germany; § Bavarian Center for Battery Technology (BayBatt), University of Bayreuth, 95448 Bayreuth, Germany; ∥ Karlsruhe Nano Micro Facility (KNMFi), 76344 Eggenstein-Leopoldshafen, Germany

**Keywords:** Solid-state batteries, void formation, lithium metal anode, *operando* SEM, microstructure, mechanical stress, reliability

## Abstract

A leap forward in the energy density of solid-state batteries necessitates the successful implementation of metal electrodes. A serious problem of metal electrodes is their nonuniform deposition and dissolution, which can lead to dendrites and voids. Here, we report on formation, growth, and shrinkage of voids based on *operando* SEM observations of lithium metal in contact with a Li_7_La_3_Zr_2_O_12_ (LLZO) electrolyte. Our observations reveal a general tendency for the replenishment of voids upon redeposition of lithium. We explain this by gradients in the mechanical stress that act as driving forces for atom motion inside the electrode and thus feed lithium away from the interface back into existing voids, which restore the original shape of the electrode. During operation, lithium atoms are inserted or extracted at the interface, creating mechanical stresses inside the electrode. In electrodes with smaller lithium grains, we observe that the shape of voids is strongly influenced by the microstructure of the lithium metal and that grain boundaries can obstruct void growth. We consider diffusion along lithium grain boundaries and the inner surface of voids as important pathways for metal transport. We suggest that the local balance of the diffusive metal fluxes is responsible for the evolution of the shape of the metal electrode.

## Graphical abstract

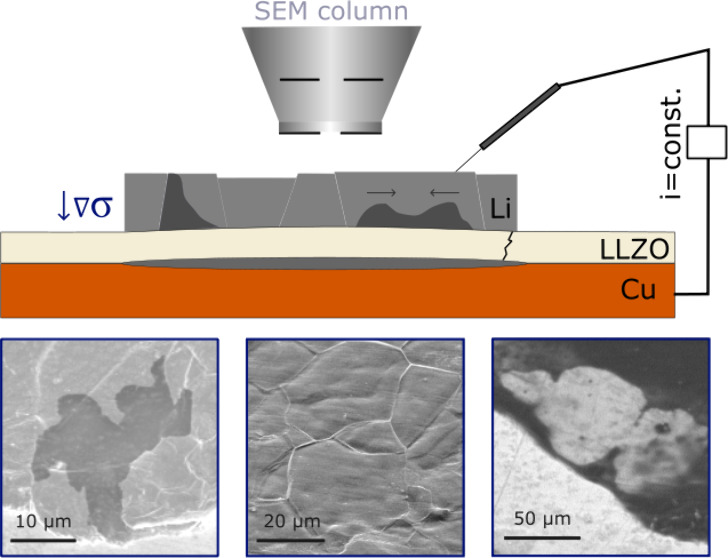

## Introduction

Solid-state batteries promise higher energy densities compared to conventional lithium-ion-batteries.^1^ This is achieved by replacing a graphite anode by lithium metal.^2,3^ So far, the implementation of lithium metal anodes in batteries with liquid electrolyte is challenging due to the inhomogeneous plating of lithium and resulting low columbic efficiency.^4^ Replacing liquid electrolytes by solid electrolytes promises enhanced safety and more uniform plating at the interface between metal and the solid electrolyte.^5^ This interface governs the rate capability and reliability of solid state batteries.^6,7^ Solid-state batteries still suffer from metal filaments^8–10^ that propagate through the solid electrolyte while plating, and formation of voids (also called pores)^11–14^ upon stripping.^15–17^ While lithium is dissolved through a solid electrolyte, the cell voltage typically increases after some time. Impedance measurements attribute this increase in interface resistance during anodic dissolution to void formation.^18^ Dissolving lithium through a solid electrolyte creates lithium vacancies at the interface. Especially at high current densities, the diffusive flux of vacancies away from the electrochemical interface is slower than the generation of vacancies at this interface, leading to a rise in vacancy concentration.^19^ These excess vacancies can accumulate and form voids in the metal electrode. Voids are not only a problem for reversible cell operation, but they also increase the interfacial resistance and lead to local current constrictions,^20^ that are held responsible for the nucleation and growth of dendrites.^21,22^

There are multiple strategies to improve the performance of solid-state batteries with alkali metal anodes, e.g., enhanced temperatures,^23–25^ increasing stack pressure,^23–25^ low current densities,^26^ pulsed current protocols,^27^ or increasing the thickness of lithium metal electrodes.^26^ While these strategies can reduce the cell resistance, they do not prevent the formation of voids. To date, further improvement of electrochemical performance is limited by a lack of understanding of the fundamental mechanisms controlling voids in lithium metal close to the interface. Due to the nature of the buried interfaces, morphological changes inside the lithium metal anode are challenging to observe.^28^ *In situ/operando* characterization combines electrochemical experiments with investigations that deliver complementary information. So far, *in situ/operando* microscopy at the anode side of solid-state batteries mainly focuses on the observation of plating of lithium (in anode-free configuration) and dendrite growth in solid electrolytes.^29^ There are a limited number of reports that study void formation at the buried interface during the operation of a cell. Several groups have studied the interface between lithium metal and the solid electrolyte using X-ray tomography.^30–33^ Lu et al. used an Li|Li_7_P_3_S_11_|In cross-section to observe void growth using a light microscope.^12^ The growth of voids in sodium using a FIB cross-section in an SEM was investigated by Fuchs et al.^34^ Their observation shows that void growth starts within individual grains, while the grain boundary stays intact.^34^ Other works also show that the microstructure of the lithium metal has a profound effect on void formation and growth.^35,36^

In this study, we investigate buried voids using SEM. We observe thin and small lithium metal electrodes that were annealed to achieve different microstructures. These lithium electrodes are located on top of thin electrolytes on copper substrates. We use LLZO as solid electrolyte due to its stability against lithium metal^37^ and the corresponding negligible charge transfer^6^ resistance. Our cells are manufactured by the deposition of LLZO films on a copper substrate using the Powder Aerosol Deposition Method (PAD or ADM).^38,39^ During our *operando* SEM investigations, we used secondary and backscattered electrons in combination with different detectors to observe changes of the lithium metal electrodes at different depths underneath the electrode surface. With our observations, we aim to contribute to a better understanding of void growth and shrinkage and to gain information on the interaction of voids with the microstructure of lithium metal.

## Experimental Methods

### Cell Materials

In our experiments, we use dense LLZO films on copper substrates manufactured at room temperature using the Powder Aerosol Deposition method (PAD or ADM). By accelerating an aerosol containing nitrogen gas and ceramic powder toward a substrate, ceramic films are deposited.^38,39^ As a result of the deposition method, the ceramic films are nanograined, with grain-sizes in the range of tens of nanometers. LLZO powder (Schott AG, Mainz, Germany) is deposited on copper substrates with a size of 7 mm × 7 mm and a height of 0.5 mm. The thickness of the deposited LLZO films is about 30 μm. We use commercial lithium foil (99.9% Lithium foil, Alfa Aesar) and reduce its thickness by rolling inside an argon filled glovebox (water and oxygen content <0.1 ppm) for electrode manufacture.

### Cell Assembly

We cut the rolled lithium and placed it on the LLZO films to create Li|LLZO|Cu cells. To ensure good contact between LLZO and lithium, the cells are clamped in a vise at a fixed distance and left at 80 °C overnight. These cells are termed cells with fine-grained (FG) lithium. To create cells with larger lithium grains, lithium was pressed on the LLZO in the same device and kept at 100 °C overnight, followed by heat treatment of the whole cell at 230 °C for 5 min. We assume that lithium metal in cells that have been annealed beyond the melting point of lithium contains a low density of defects and exhibits larger grains. These samples are termed cells with coarse-grained (CG) lithium. The cell preparation for all experiments is summarized in Table S1 in the supplement.

### Operando SEM Observations

We observe lithium metal electrodes during dissolution and deposition inside an SEM (Merlin, Carl Zeiss AG, Oberkochen, Germany). Figure 1a shows the experimental setup. The cells are mounted on a 45 ° SEM holder and transferred under argon to the SEM using a transfer system (EM VCT 100, Leica, Wetzlar, Germany). We used a micromanipulator (Kleindiek, Nanomanipulator MM3A, Reutlingen, Germany) to contact the lithium metal electrodes in the SEM.

**Figure 1:**
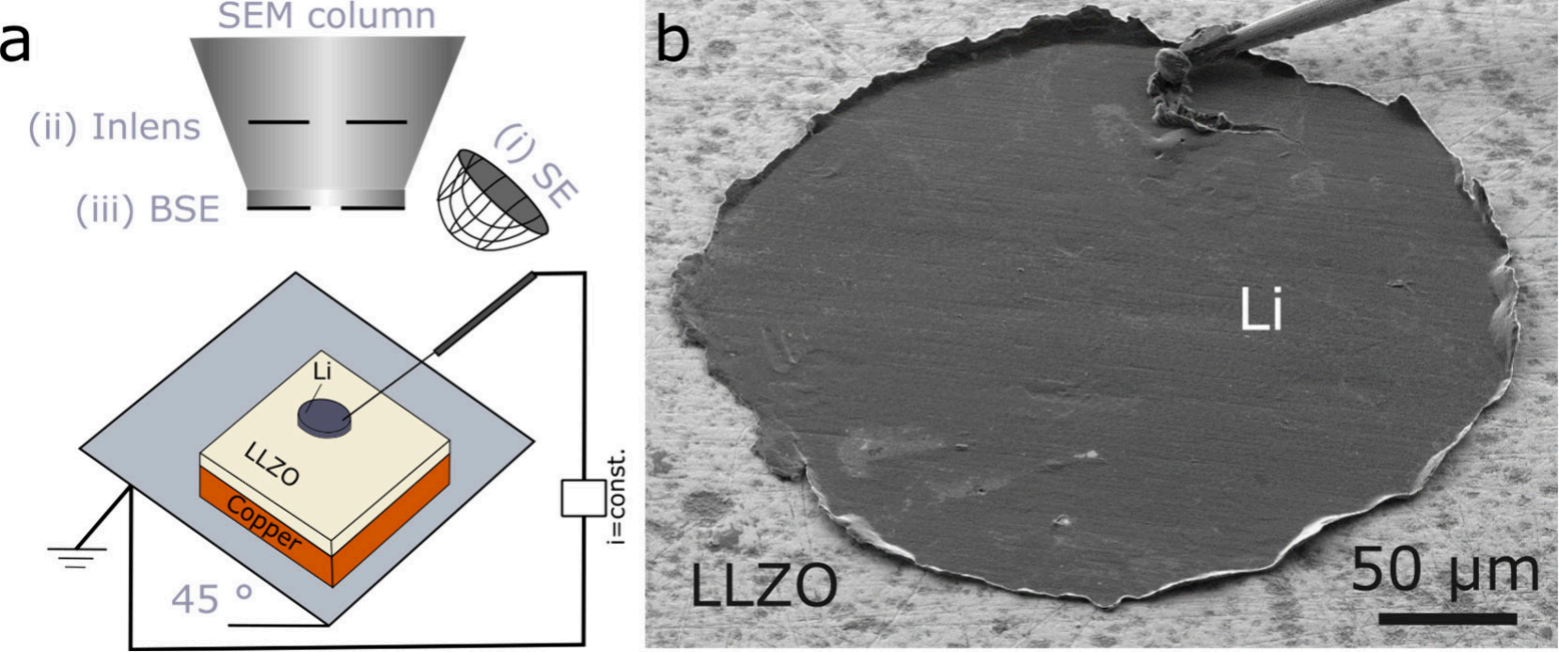
(a) Experimental setup. (b) Overview of an exemplary electrode in its initial state.

Figure 1b shows a typical cell where a lithium electrode on an LLZO film is contacted by a tungsten needle. We used different imaging conditions in the SEM to investigate lithium dissolution and redeposition. For the incoming electron beam, we use either 3 keV or higher energies (12, 18, or 30 keV) to change between different penetration depths into the Li|LLZO layers. We use different detectors for the generated electrons, as illustrated in Figure 1a: (i) A conventional Everhart-Thornley detector (SE) to detect electrons up to 50 eV to obtain images of the surface, and (ii) an in-column semiconductor detector (Inlens) with enhanced efficiency for low-energy secondary electrons, and therefore, improved surface contrast. The Inlens detector does not have an energy threshold and detects electrons with higher energy up to the accelerating voltage, which originate from deeper regions within the sample (backscattered electrons). Therefore, the Inlens images at 3 keV are an overlay of detailed information from the surface and volume of a sample. For example, this detector can be used to image voids that are located directly underneath the surface that appear as darker regions in the images. We use a third detector, (iii) a four-quadrant backscatter detector (BSE) mounted underneath the pole piece for elastically backscattered electrons with energies beyond 5 keV (Figure 1a). These images contain little to no surface information and are useful to image deeper into the electrode volume. In most experiments, images were recorded with both the SE and Inlens at 3 kV every 5 min. A subset of our data was imaged with backscattered electrons at 18 kV and recorded every 10 min.

### Electrochemical Characterization

We conduct electrochemical measurements of Cu|LLZO|Li cells using a potentiostat (CompactStat.e, Ivium Technologies B.V., Eindhofen, Netherlands). All experiments are run under galvanostatic conditions. We used a low current density of 20 μA/cm^2^ to observe the dissolution of lithium in detail. All experiments start with dissolution of the lithium electrode. While the pressed metal electrode dissolves, lithium is plated into the copper-LLZO interface, generating symmetrical cells.

### Helium Ion Microscopy and Preparation of Cross Sections

At the end of our experiments, lithium metal was additionally imaged using a helium-ion microscope (Orion Nanofab, Carl Zeiss AG, Germany). A neon gas pressure of 2 × 10^6^ mbar and an acceleration voltage of 25 kV was used to generate the beam. Cross sections were fabricated by a neon-ion beam using a dose of 8 nC/cm^2^ and a dwell time of 2 μs. Imaging was performed by helium-ions. The samples were transferred between our glovebox and the microscope under argon.

## Results

The results presented here are based on *operando* SEM experiments that investigate the effect of lithium metal microstructure on the electrochemical deposition and dissolution of Li through LLZO. Experiments have been performed on 17 cells, resulting in a multitude of observations. In the following, we categorize our observations and describe mechanisms and dependencies that we consider representative for the behavior of cells containing a metal anode and solid electrolyte. The supplement contains videos with additional data of the experiments that are presented in the figures. The complete set of data in the form of videos can be found online.^40^

Figures 2a-d shows the images of a coarse-grained (CG) lithium metal electrode before and after galvanostatic dissolution over ∼320 min. The SE images (Figures 2a and 2b) exhibit only minimal differences in the lithium metal surface despite ongoing dissolution. Images taken with the BSE detector (Figures 2c and 2d) contain information about the inside of the lithium metal electrode. At 12 keV and beyond, most of the outgoing electrons are generated within the LLZO layer and are attenuated by the lithium located on top. Therefore, the gray levels in these images correlate with the thickness of the lithium metal electrode. We attribute the brighter areas within the electrode in Figure 2c and 2d to the formation of voids. With ongoing dissolution, the round voids grow and coalesce. The corresponding electrochemical data are shown in S3.

**Figure 2:**
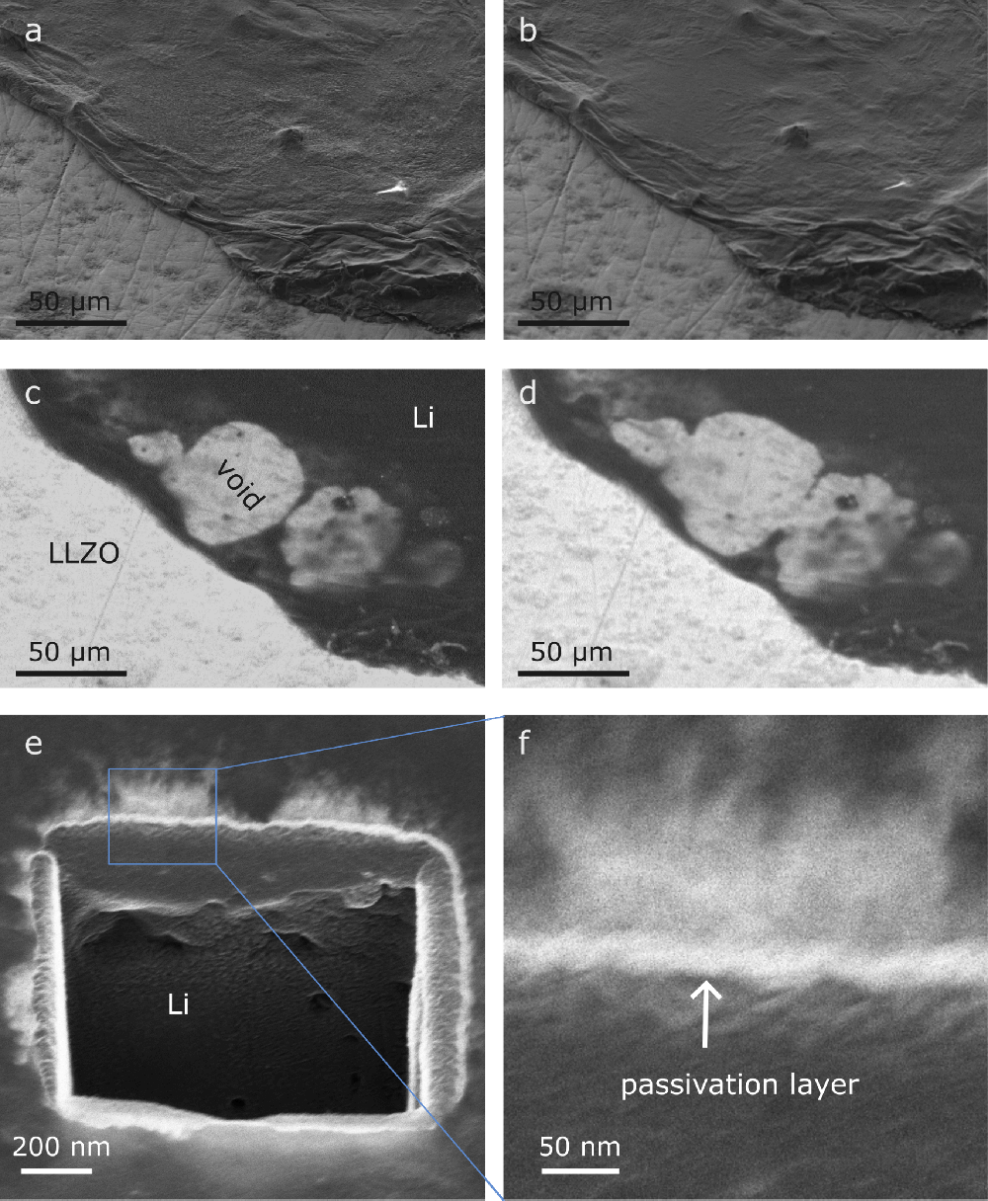
(a), (b) SE images (3 kV) of the surface of a CG-electrode before and after the dissolution for ∼320 min. (c, d) BSE images (12 kV) recorded at the same location and same states of charge as 2a and 2b, respectively. (e) HIM image of a cross-section of a lithium electrode and (f) the passivation layer on the lithium at higher magnification.

Figures 2e,f shows a cross-section in the lithium metal cut with neon-ions and imaged with helium-ions. A 10–20 nm thick layer on the lithium metal is visible. This passivation layer forms despite careful sample preparation and transfer to SEM under argon atmosphere. We assume this layer to be present in all experiments. In our experiments, the passivation layer did not disappear during dissolution (Figure S2). The layer appears to be mechanically stable while dissolving lithium. In Figures 2a,b the growth of the pore inside the metal electrode is not visible at the electrode surface.

Figure 3 presents observations of the dissolution and subsequent plating of lithium on CG lithium metal. This observation demonstrates that a high degree of reversibility of dissolution and redeposition is possible. The electrochemical data in Figure 3a are recorded on the entire electrode and contain the dissolution and redeposition of lithium of the same amount of charge (OCV of 2 h in between). The BSE images in Figure 3 show a section of the electrode, with a single void growing close to the edge of the electrode. The first BSE image of the image series was recorded after sufficient deposition onto the reservoir-free counter electrode, and a void was present in the observed region of the working electrode. Figure 3b contains labels describing the different regions in the BSE images. The first row of images (Figures 3c-f) shows the growth of this void, and the second row (Figures 3g-j) displays the subsequent refilling. Each column of Figure 3c-j was recorded at the same state of charge, the upper row during dissolution, and the second row during redeposition. When lithium is redeposited, the pore shrinks from the outside toward the inside in similar, but reverse order compared to pore growth. Video S1 shows the full data set of the experiment, including the initial state of the lithium electrode. A video of the entire electrode with more voids that show similar behavior can be found on KITopen^40^.

**Figure 3:**
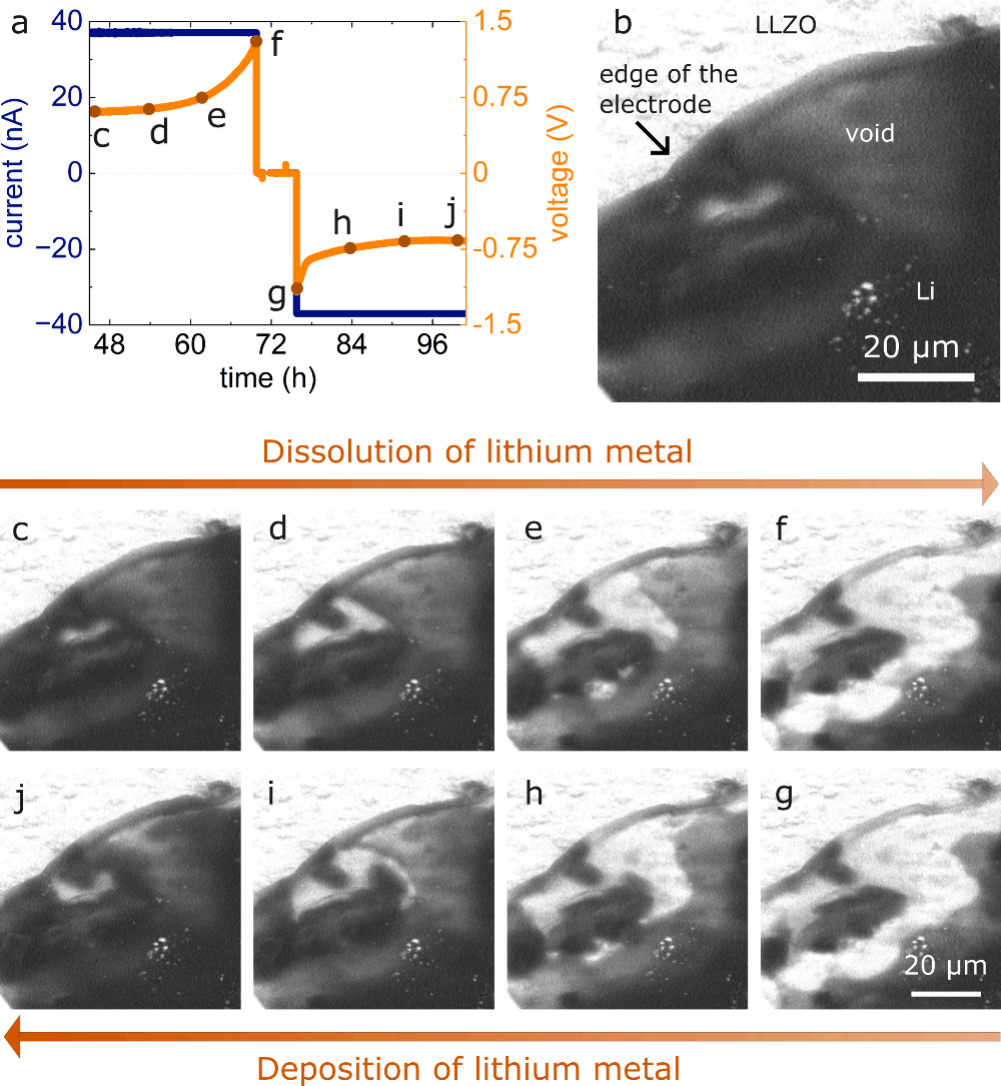
Void growth and replenishment in a CG lithium electrode with the same dissolution and redeposition charges. (a) Voltage and current profile corresponding to a current density of 20 μA/cm^2^. BSE images of CG lithium at 18 kV: (b) Overview of the observed area, same as in image (c). (c)–(f) growth of the void during dissolution of lithium. (g)–(j) Replenishment of void during deposition of lithium. Subfigures (c)–(g) were recorded at the same magnification. Video S1 shows the full experiment with the initial growth of the void and the replenishment during the redeposition of lithium.

The degree of reversibility decreases when voids interact with each other. Figure S4 and Video S2 show a different region within the same electrode as in Figure 3, in which three voids coalesce to form a larger void during dissolution. Reversing the current leads to a shrinkage of this larger void, but there is no transition back to the three separated voids.

In contrast to CG lithium metal electrodes (Figures 2a, b), we observe several changes on the surface of fine-grained lithium (FG) electrodes during dissolution. Figures 4a–d and the corresponding Video S3 show a section of the surface of a FG lithium metal electrode during dissolution using an SE detector and an acceleration voltage of 3 keV. Figure 4a shows the initial state of the electrode. Fine parallel lines oriented close to horizontal in Figure 4a are a result of the deformation during preparation and are caused by pressing the lithium on the LLZO. With ongoing dissolution, a structure appears at the surface and develops as shown in Figures 4b-d. The structure consists in local changes of the thickness of the electrode and bright lines at the surface that bear resemblance to typical metal microstructures. Other groups related these lines to grain boundaries in the lithium metal,^34^ and therefore, we interpret the bright lines as grain boundaries. During dissolution and evolution of the electrode surface topography, the preexisting parallel fine lines (artifacts from sample preparation) disappear. The appearance of grain boundaries on the surface of the electrode is not observed in the cells with CG lithium electrodes (e.g., Figure 2). There is a local maximum in the voltage data in Figure 4e at the very beginning (∼100 s). We attribute this maximum to the nucleation of lithium on the reservoir-free side. Despite the strong changes observed by SEM, there is only a small increase in voltage in Figure 4e. Figure 4f is an overlay of Figures 4a-d that were inverted in their grayscale to emphasize the lines. The observed lines (grain boundaries) do not significantly move during the stripping process (Figure 4f).

**Figure 4:**
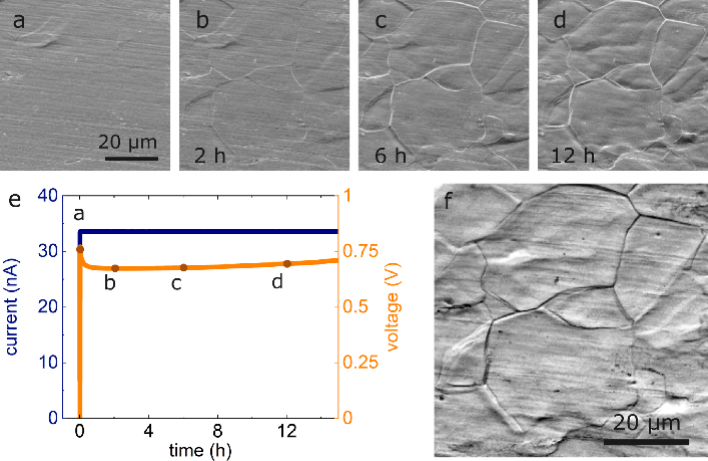
(a)–(d) SE images (3 kV) of the surface of a FG lithium metal electrode during dissolution for 12 h. (e) Electrochemical data of the experiment using a current density of 20 μA/cm^2^. (f) Overlay of Figures 4a–d, inverted in color, to visualize the evolution of the lithium microstructure. Subfigures (a)–(d) were recorded at the same magnification. Video S3 shows the evolution of the electrode surface.

We observe that grain boundaries in the Li metal can both suppress and enable pore growth and propagation. Figure 5 reports the growth of a void using the Inlens detector and 3 keV beam energy. Using this imaging condition, the void appears darker compared to the surrounding lithium. The void growth is anisotropic and influenced by the microstructure of the Li metal. In Figure 5a, on the right side, the void adjoins a grain boundary, marked by a red cross. The void appears to be pinned. The grain boundary represents an obstacle for void expansion, and consequently, the void does not cross this grain boundary with ongoing dissolution (Figures 5b-c). At its left side, the void is in contact with another grain boundary, marked by a red cross in Figure 5b. Here, the local growth of the void is restricted by this grain boundary (Figures 5a, b), and the void grows along the grain boundary. With further dissolution, the void eventually propagates across this grain boundary (green arrow in Figures 5c).

**Figure 5:**
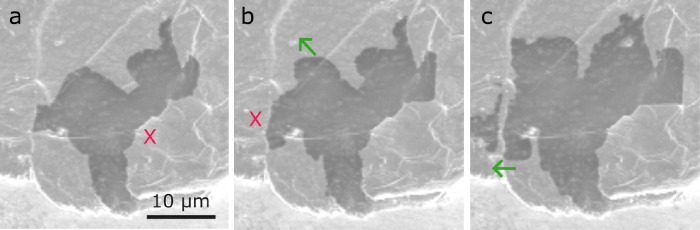
(a)–(c) Inlens images (3 kV) of the interaction of a void with different grain boundaries. Subfigures (a)–(c) were recorded at the same magnification. Figure S5 shows the corresponding electrochemical data. Video S4 shows the growth of this void.

In the absence of grain boundaries, some voids exhibit symmetry in their shape. Figure 6 displays the growth of a void with a rectangular shape in the center of an individual grain. The angles of the sidewalls of the void are close to 90 °. Figure 7 shows the evolution of a void during an OCV period of 12 h. The void changes shape and develops facets on its surface.

**Figure 6:**
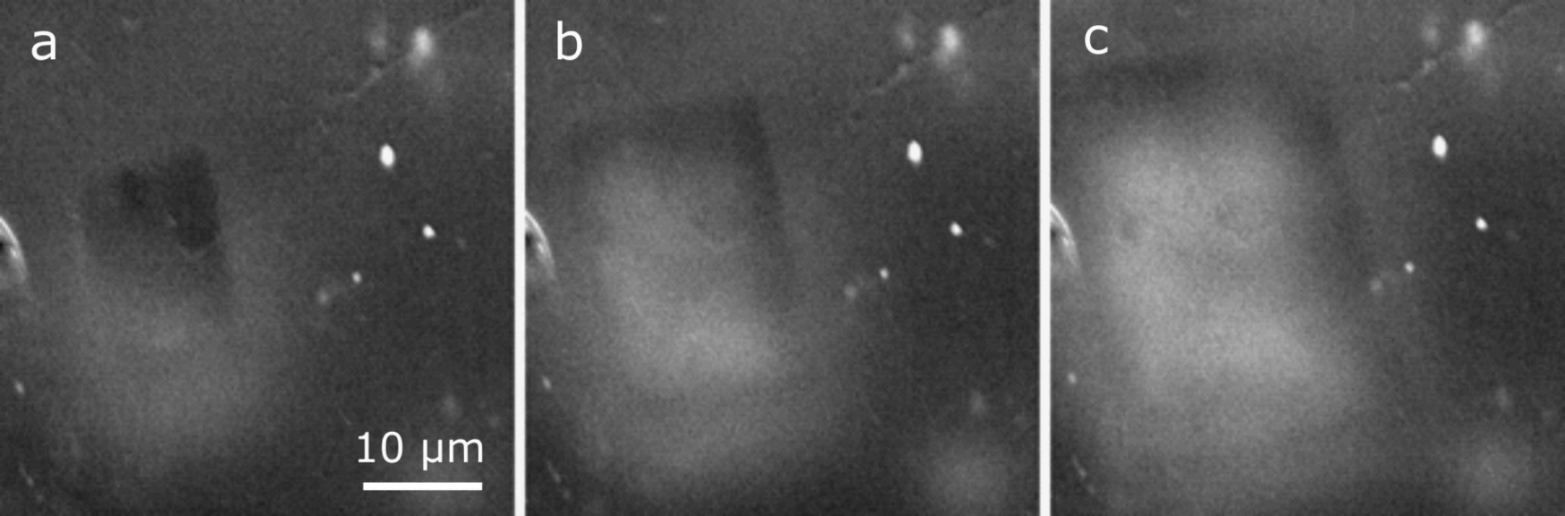
(a)–(c) SE images (18 kV) of a void with rectangular shape. Images are recorded at 45 ° tilt and corrected for zero tilt. Subfigures (a)–(c) were recorded at the same magnification. Figure S6 shows the corresponding electrochemical data. Video S5 shows the evolution of this pore.

**Figure 7:**
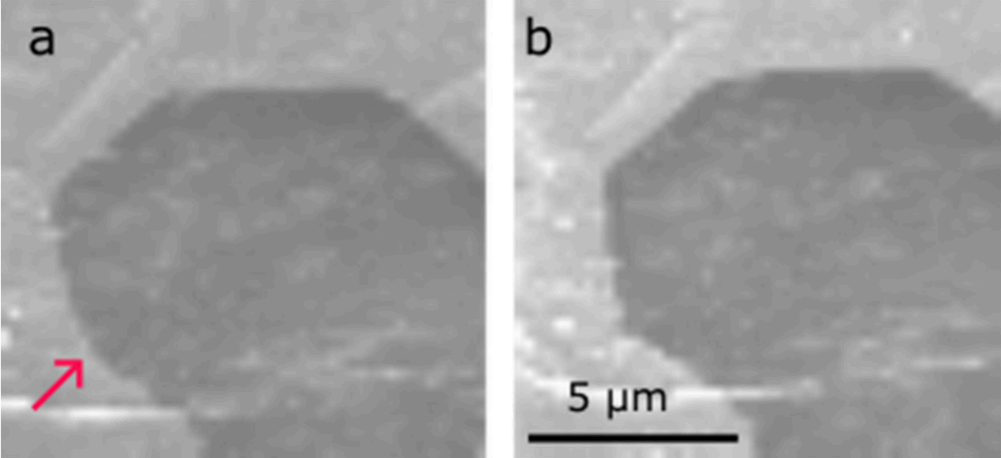
(a), (b) Inlens images (3 kV) of the evolution of a void in a CG electrode and the emergence of facets during an OCV period of 12 h. Subfigures (a), (b) were recorded at the same magnification.

Several electrodes develop a partial detachment from the LLZO during dissolution. Typically, a thin area along the electrode perimeter detaches from the electrolyte and bends away from the electrolyte surface as seen in Figures 8, S8, and Video S6. Figure 8a shows the initial state of the lithium electrode, while Figure 8b shows the lifted and tilted edges of the electrode. The detached edges of the electrode do not have direct contact with the LLZO. The red arrow marks a void that starts to grow within this detached region. Figures 8b-d show the progression of the void growth. Another example of a detached electrode edge can be found in Figure S8. Here, a void grows from the center of the electrode into the lifted edge. A void grows in a part that is detached from the electrode. The early stage is marked by a red arrow. The refilling of this void and a second growth are available in Video S7. Figure S7 shows the corresponding electrochemical data.

**Figure 8:**
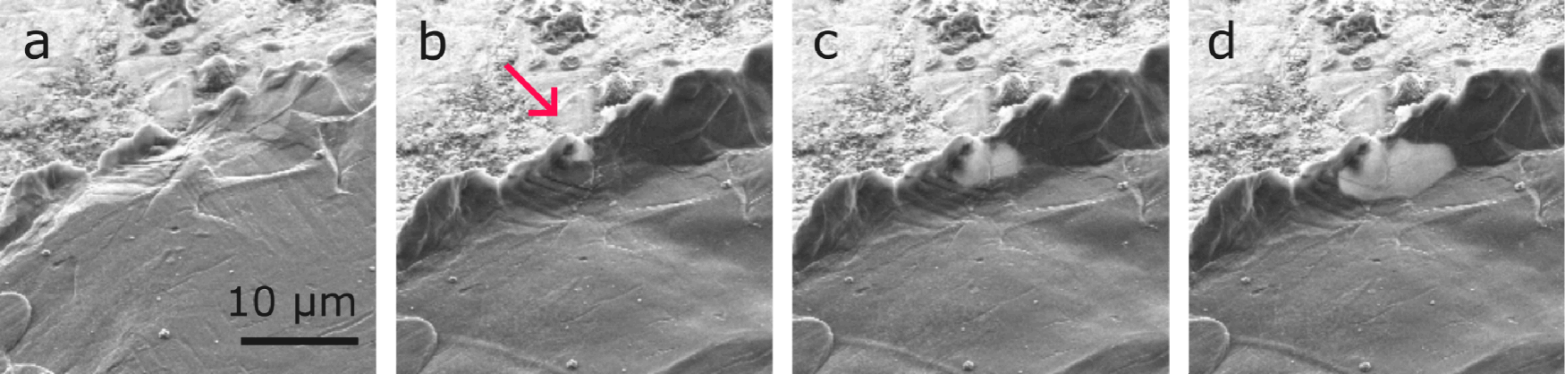
SE images (3 kV) of the edge a FG lithium metal electrode. (a) Initial state. (b)–(d) Void growing into detached edge of the Li electrode. Subfigures (a)–(d) were recorded at the same magnification. Figure S7 shows the corresponding electrochemical data. Video S6 shows the detachment of the electrode from the electrolyte surface. The refilling of this void and a second growth is available in Video S7.

We observed cracks in LLZO and their influence on voids. Figures 9a and 9b are images of the CG Li electrode recorded after different amounts of dissolution of lithium. Voids grow close to the edge of the electrode. In the regions with voids, the surface of LLZO becomes visible, revealing a curved crack in the solid electrolyte. The crack is roughly concentric to the perimeter of the electrode as can be seen from the 30 kV BSE image in Figure 9c. Figures 9b and 9c were recorded ∼320 min later than in Figure 9a. These images demonstrate that that despite the underlying crack, dissolution continues and voids continue to grow. In several cells, we observed that cracks in the electrolyte do not lead to short circuits.

**Figure 9:**
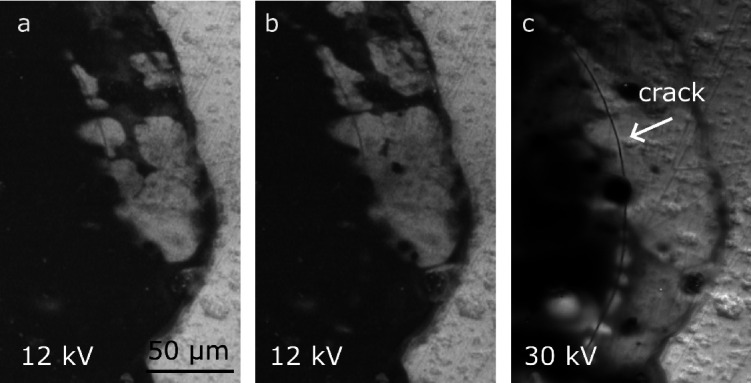
Growth of voids on top of a crack in the LLZO films. (a) BSE image (12 kV) of a CG electrode. (b) 320 min of additional dissolution. (c) BSE image (30 kV) at the same state of charge as in Figure 9b. Subfigures (a)–(c) were recorded at the same magnification. Electrochemical data are listed in Figure S3.

## Discussion

We electrochemically and microscopically investigate the dissolution and deposition of lithium metal through LLZO on 17 lithium metal electrodes with two different microstructures. SEM imaging with different acceleration voltages and detectors was used to image the surfaces and reveal the buried interfaces of the growing and shrinking metal electrodes. The variety in the evolution of the shapes of the electrodes suggests that the deposition/dissolution of metal through the solid electrolyte is affected by several underlying mechanisms. Based on our observations, we categorize the phenomena and discuss the underlying mechanisms that we consider important for the operation of ASSBs with metallic anodes.

This study makes use of a ∼30 μm LLZO electrolyte layer produced by powder aerosol deposition at room temperature, which leads to a nanocrystalline microstructure.^38,39,41^ Our lithium electrodes have lateral dimensions of the order of 100 μm (about 3 orders of magnitude larger than the grains of the electrolyte). Consequently, no inhomogeneity of the electrolyte becomes apparent in the experiments.

In all experiments, a passivation layer was present on the lithium metal. This is a common phenomenon on lithium metal that cannot be avoided even in ultrahigh vacuum.^42^ In a comprehensive study, Otto et al. determined the chemical composition of various lithium samples using XPS and Tof-SIMS. They showed that this layer on lithium that was stored in an argon-filled glovebox typically consists of Li_2_O, LiOH, Li_2_CO_3_, and Li_3_N.^43^ In the FG electrodes, bright lines emerge in this surface passivation with ongoing dissolution (Figure 4). Such lines in the SEM images have been observed by other research groups and were attributed to the grain structure of lithium metal.^35,44,36^ Fuchs et al. confirmed using EBSD that most of the bright lines correspond to grain boundaries in the lithium.^34^ We also attribute the bright lines in our experiments in FG electrodes to the lithium grain boundaries. The size of grains of commercial lithium foils are often in the range of several hundred micrometers.^45^ Our grains are significantly smaller (e.g., Figure 4), due to rolling during sample preparation. In the CG lithium, we do not observe bright lines (Figures 2 a,b). For these cells, we assume that the grain size is in the range of the size of the electrode.

### Void Evolution within an Individual Lithium Grain

Void formation and filling within individual grains can be reversible, which is apparent in the similarity of the two rows in Figure 3. At the same state of charge, the porous electrode adopts a similar shape (ideally the same shape) during charge and discharge. Any deviation from this forward–backward shape symmetry of charge and discharge (both rows in Figure 3 are very similar) can be associated with irreversibility and consequently degradation.^18,32^ Figure S4 shows three voids that interact and coalesce^46^ to form a larger void. In this case, the local shape of the electrode changes irreversibly, very likely associated with degradation upon further cycling. We expect an improved cycle life for metal electrodes in which the growing and shrinking voids do not interact. The reversibility in shape presented in Figure 3 was observed for several isolated voids. Filling of voids during redeposition was not expected for our cells that operate without stack pressures since the inner part of voids is electronically isolating and charge transfer between electrons and ions, and consequently, lithium deposition cannot take place there. Plating is possible not only directly at the very narrow perimeter of a void, where electrons are available, but also at any point of the Li-LLZO interface. Uniform deposition into the interface without increasing the contact area would lift the remaining lithium as a whole, thereby elongating the existing voids perpendicular to the surface of the electrolyte (Figure 10a). This mechanism results in pillar-like structures of lithium. In contrast, we observe that the voids refill from the outside to the inside (Figure 10b), which is advantageous with respect to the reversibility of electrode shape and charge. The voltage data (Figure 3a) also indicate an increase in contact area while plating. Our observations are in agreement with simulation by Zhang et al., where they predict an increase in contact area for low current densities and columnar growth morphology for higher current densities.^47^

**Figure 10:**
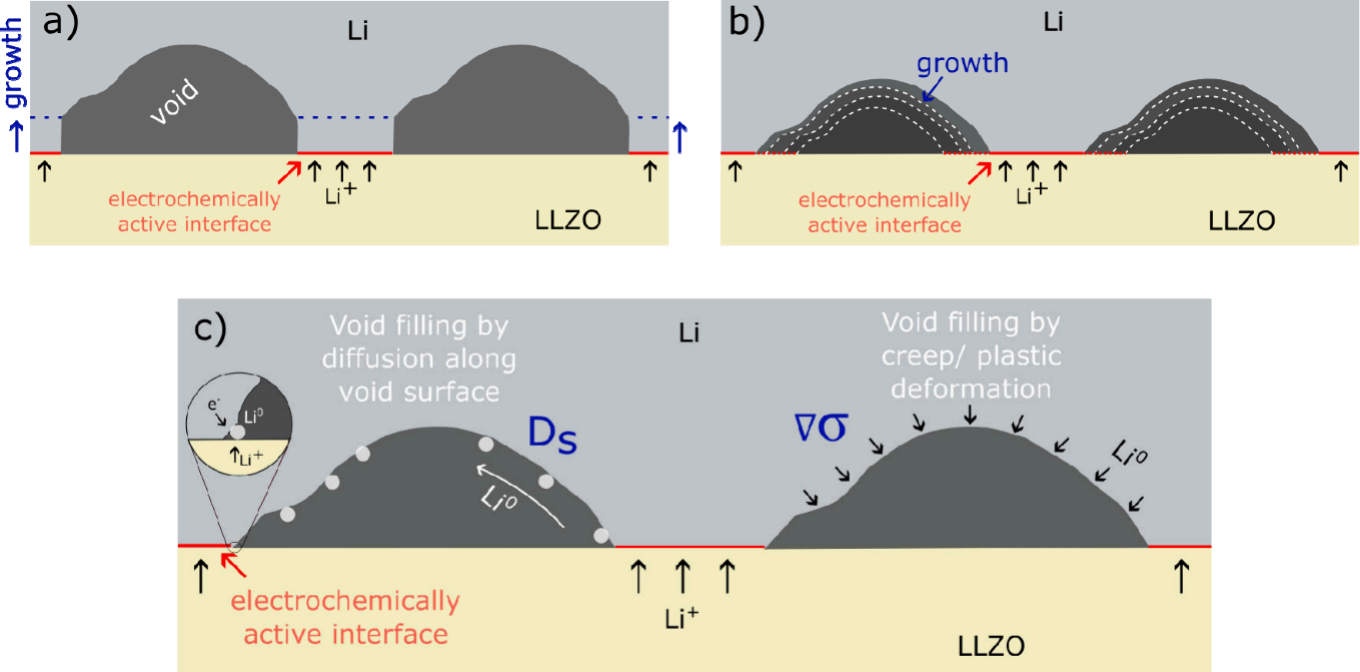
Two fundamentally different mechanisms of lithium deposition: (a) Lithium insertion into the interface and local growth without refill. (b) Refill of existing voids. (c) Two different mechanisms for the refill of voids.

In our experiments, we observed a variety of different shapes of voids. In a few cases, we found voids with highly symmetrical rectangular shape (Figure 6) or voids that appear to have a tendency for right angles between some of their edges (e.g., upper right in Figure 5c). We suggest that the shape of these voids reflects the symmetry of the lithium crystal with its body-centered cubic (bcc) structure. This rectangular shape can be explained by the minimization of surface energy of the sidewalls of the void, in which the surface with the lowest energy—which typically is a low index crystal plane—grows at the expense of other surface orientations. The angles of 90 ° in Figure 6 imply that the orientation of the lithium grain perpendicular to the electrolyte interface is either [100] or [110]. Periodic density functional theory calculations identify the (110) plane in Li as the one with the lowest energy per surface atom.^48^ In an *in situ* TEM experiment, selected-area diffraction was used to identify the surface of a void as a (110) plane.^49^ When a metal flux is present, i.e., during dissolution, the shape of the void is governed by the different diffusivities of the individual orientations of the surfaces of the sidewalls. Once the current is turned off and the mechanical stresses in the electrode equilibrate, diffusion kinetics does not govern the shape of the void anymore. Then voids relax toward their thermodynamic equilibrium, and their faceting increases (Figures 7b).

### Voids and the Effect of Lithium Microstructure

The behavior of voids as discussed in the previous section changes in the vicinity of grain boundaries in the lithium. The polycrystalline microstructure of the FG electrodes leads to more complex shapes of voids, which are likely to reduce the reversibility of plating and stripping. The evolution of the void in Figure 5 shows that its interactions with different grain boundaries can vastly differ. Either a grain boundary is penetrated by the void (green arrows in Figure 5) or a grain boundary restricts its growth (red crosses in Figure 5). Grain boundaries change the orientation of the crystal lattice between two neighboring grains. Numerous configurations between two neighboring grains are possible, and five parameters are required to identify a grain boundary. In contrast to this rather complex description, strongly simplified approaches characterize grain boundaries by their energy or their kinetic properties, i.e. their diffusivity, *D*_GB_. The surface energy of the lithium on the void walls, γ_S,_ is around 500 mJ/m^2^.^48^ This is higher than the energy of grain boundaries of lithium, γ_GB,_ which is below 200 mJ/m^2^.^50^ With these energies, an exchange of boundary energy with surface energy is not beneficial, and voids will not align along former boundaries. Based on the energies, we expect growing voids to be hardly influenced by the presence of grain boundaries with respect to their shape and their speed of growth. Figure 5 shows that voids can interact with boundaries. Therefore, instead of energy, we suggest that diffusional mass transport governs the interaction between voids and GBs in Figure 5. At a void in contact with a grain boundary, at least two diffusional pathways are active, namely, diffusion along the void surface, *D*_S,_ and through the grain boundary, *D*_GB_. As known from creep of metals, the local growth of a void depends on the net flux to/from a site, i.e. the flux divergence.^51^ When there is a net flux of lithium atoms away from the void, it locally grows; otherwise, it shrinks. The observation in Figure 7 demonstrates that lithium is redistributed along the walls of voids at least over distances of several micrometers. In the proximity of a growing void, the diffusional fluxes along its surfaces and the neighboring grain boundaries interact. Our observation that the bright lines of certain grain boundaries transition into void walls and remain stable during void evolution suggests that around these locations (sites marked by a red cross in Figure 5) there is no net diffusional flux. Lithium grain boundaries that carry low fluxes contribute less to the net flux of atoms and may not strongly affect the evolution of the shape of the void (sites marked by the green arrow in Figure 5). Our observations are related to the ones by Fuchs et al. on sodium metal electrodes, who observed that voids grow inside grains while the neighboring grain boundary stays intact.^34^ Besides kinetic effects around grain boundaries, contamination of grain boundaries, i.e., the presence of elements like oxygen or nitrogen, may play a role by altering the chemistry and prevent dissolution of the lithium-containing chemicals (e.g., Li_2_O, Li_2_O_2_, Li_3_N). Cells in applications might experience the same phenomenon. The presence of bright lines in the micrographs at the sample surface that have been identified to correlate with GBs supports the existence of impurities. The higher yield of secondary electrons in the SEM image at these locations may originate from elements that are heavier than lithium. Within the experiments presented here, the clear distinction between microstructure and chemical effects is not possible. The influence of the passivation layer on void kinetics cannot be excluded, but it is hard to envision how a passivation layer located at the outer surface can block the motion of an underlying void.

The evolution of the top surface of the lithium metal is influenced by the microstructure of lithium, as can be seen in Figure 4. Dissolution of lithium is inhomogeneous and different grains sink in by different amounts. Since metal dissolution can only happen where charge transfer takes place, i.e. at the LLZO-Li interface, two possible mechanisms can be identified that can explain the observed grain-dependent changes in height. First, the anisotropic, orientation-dependent dissolution of different grains at the LLZO interface and simultaneous sliding of these grains down to the interface to maintain contact. This could avoid high tensile stresses at the interface and locally prevent the nucleation of a void at the interface. Second, the diffusion of vacancies away from the interface toward the surface of the lithium metal. Since the diffusion along grain boundaries is much faster than through the bulk metal, grain boundaries would be the most probable transport path for vacancies/atoms.^50^

### Diffusion Pathways in Lithium

Lithium melts at 453.7 K and room temperature is already 65% of this melting temperature. Compared to most metals encountered in daily life, the homologous temperature of *T*_H_ = 0.65 *T*_M_ is very high, and therefore, typical high temperature deformation pathways such as creep are active. Deformation mechanism maps for alkali metals^52^ and lithium^53^ show that grain boundary diffusion, *D*_GB,_ (Coble creep) is dominant over volume diffusion, *D*_V,_ (Nabarro-Herring creep). At 0.65 *T*_M_, most metals exhibit high surface diffusivities, *D*_S_, sometimes greater than *D*_GB_. This is also the case for lithium: Simulations of lithium show that surface diffusivities^48^ and grain boundary diffusivities (between ∼10^–7^ and 10^–6^ cm^2^/s)^50^ at RT are more than 3 orders of magnitude higher than simulated and measured values of *D*_V_ (10^–11^ cm^2^/s).^54^

In our experiments, the changes of the electrode surface were observed at distances (up to ∼20 μm) from the electrochemical interface, where vacancies are generated. In Figure 8, a void forms inside a lifted edge of the lithium electrode in a part that has no ionic connection to the electrolyte indicating that long-range metal transport takes place. Since *D*_V_, i.e., the diffusion of vacancies within the grain interior, is slow, other diffusion mechanisms must be active. Good candidates for faster metal transport are grain boundary diffusion, *D*_GB,_ and surface diffusion, *D*_S_. The presence of long-range metal diffusion within the electrode in Figure 8 may also account for the uneven surfaces on polycrystals in Figure 4 that may form by atoms diffusing from the top of the electrode to the interface rather than sliding individual grains. Lithium metal electrodes always contain a passivation layer at the surface (Figure 2e, f). Based on this observation, in Figure 8, interface diffusion, *D*_I,_ underneath the passivation layer, may also play a role as a path for lithium atoms. To our knowledge, values for *D*_I_ are not available, and therefore, the role of interface diffusion via the passivation layer remains unclear.

Long-range metal transport can be accomplished not only via the motion of individual vacancies but also via the motion of voids. The voids exhibit an inner surface on which lithium adatoms can diffuse very fast (*D*_S_). In this process, atoms move fast from one side of the void to the other side, thereby translating the void against the flux of atoms. Figure S9 illustrates this transport pathway. This void-based mechanism allows for facile metal transport and does not strongly rely on other crystal defects, such as grain boundaries or dislocations. The resolution of our microscopy in time and space might not be sufficient to observe nanovoid-based transport; nevertheless, it seems plausible that during dissolution, vacancies as well as nanovoids are vehicles that carry lithium from the electrode surface to the electrochemically active interface. Beside mobility, a driving force is required to cause a flux of atoms. Since the electrode is metallic, we expect that the same potential is present at any point, and an electrochemical driving force can be excluded. Differences in the concentration of metal atoms/vacancies, i.e., mechanical stresses, as explained in the following sections, are the only driving force that can explain the observed effects.

### Mechanical Stresses

Assuming that the vacancies in lithium are in thermodynamic equilibrium, the concentration of vacancies, *c*_*v*_ follows an Arrhenius law with temperature *c*_*v*_ = exp (– *E*_f_/*kT*) with *E*_f_ the energy of formation as the activation energy, *k* the Boltzmann constant, and *T* the temperature. A vacancy-atom pair requires more space than one atom alone, and therefore the formation energy can be reduced or increased by the presence of mechanical stress, so that the vacancy concentration under the presence of mechanical stress becomes *c*_*vσ*_ = exp­(−(*E*_f_ – σΩ)/*kT*) = *c*_*v*_ exp (σΩ/*kT*), with the hydrostatic mechanical stress, σ (tensile stresses are positive values), and the atomic volume, Ω. This means that for a fixed temperature, the vacancy concentration directly translates to mechanical stress and vice versa. Tensile stresses correspond to an increased local vacancy concentration and compressive stresses to a decrease in local vacancy concentration. The differences in their concentration, i.e., gradients of the mechanical stress, are the driving force for diffusional motion of vacancies/atoms (stress migration).

We assume that a flux of vacancies along a stress gradient leads to the growth of the void, as shown in Figure 8. Since this void is ionically disconnected from the electrolyte, only metal diffusion driven by a mechanical stress gradient can explain its growth and subsequent shrinkage. The dissolution of lithium atoms at the lithium-LLZO interface generates vacancies and therefore tensile stresses. The far end of the lifted edge of the sample is expected to be free-standing and can elastically relax its internal mechanical stresses. The stress gradient between the edge of the electrode and the interface drives the vacancies toward the edge, i.e., atoms from the edge to the interface. When the current is reversed, the same void shrinks (Video S7). Excess atoms are electrochemically generated at the interface; i.e., compressive stresses evolve, and atoms will flow from the interface to the unstressed void.

Based on our microscopy results (e.g., in Figure 3), it is not straightforward to infer the mechanism of insertion of lithium into the voids when they shrink. Two different mechanisms are plausible. First, lithium atoms may be directly electrodeposited onto the sidewalls of the void at its intersection with the LLZO. Subsequently, the atoms may redistribute by quickly diffusing along the inner surface of the void to fill it (left void in Figure 10c), thereby increasing the contact area between lithium metal and the solid electrolyte.^49^ This mechanism can encapsulate a void and lead to occluded voids.^14,55,56^ Second, the electrodeposition of lithium at an interface distant from a void generates compressive stress. This causes a stress gradient between the interface and the surface of the void that drives metallic lithium into the void by diffusion (right void in Figure 10c). The surface of the void may experience little stress, and just as in Figure 8, the flux of lithium metal away from the compressed interface may feed the shrinking void. This second mechanism can explain the refill of the void in a detached part of the electrode (Figure 8, V6, 7). Depending on the location and the ionic connection of the void, both mechanisms may act simultaneously and can close voids in the electrode.

We suggest that the observed passivation layer^42,43^ on top of the voids helps in retaining the shape of the electrode during redeposition. Shape retention of lithium structures by the passivation layer has been observed before by TEM observations.^57^ Figure S10 shows an example in which the lithium is locally fully depleted underneath the passivation layer and later forms again. The layer acts as a barrier for metal migration during void shrinkage so that the electrode retains its initial shape after voids disappeared. The passivation layer may be able to transport lithium along its inner surface to redistribute lithium inside the voids and the layer seems to withstand/carry mechanical loads. In our observations, the passivation layer and subsequent related effects are ubiquitous. The images of the electrode in Figure 4 demonstrate that the passivation layer can change its shape during the operation of the cell, for example, reshapes and moves with shrinking (Figure 4) and growing grains. This behavior can only be explained by a layer that either continuously reforms or is able to withstand large plastic deformations.^58^ Although nonmetallic, inorganic materials such as Li_2_O, Li_2_O_2_, and Li_3_N, that are contained in the passivation layer,^42,43^ are typically considered to be brittle, nanocrystalline or amorphous phases of these materials may deform plastically.^58^

Some electrodes detached from the electrolyte at their perimeter and later bent away from the LLZO surface (e.g., Figure 8). This happened only in a few samples and the origin of this effect may be related to the poor wetting of LLZO with lithium^59^ and variations in our sample preparation (electrolyte cleaning, pressing of the lithium). We suggest that lifting the electrode is a purely mechanical effect. The ionically connected middle region of the electrode sinks faster than the outer ionically detached perimeter. This change in geometry leads to a tensile stress in the surface of the lithium electrode that bends the detached outer region of the electrode upward.

Mechanical effects also originate from the reservoir-free side of our cells. Figure 9 shows a crack in the PAD-LLZO layer. Our lithium electrodes had dimensions on the order of 100 μm, while the LLZO and the underlying copper current collector were several millimeters in size. The dissolution of lithium from the small electrode and its localized deposition between LLZO and copper generate compressive stress in the freshly deposited lithium. This localized hydrostatic stress between the thick copper current collector and the thin LLZO layer leads to local bending of the thin LLZO layer depending on the shape of the deposited lithium. The mechanical stress leads to fracture of the LLZO. Based on the measured charge corresponding to Figure 9a, a nominal thickness of lithium of 9.6 μm was deposited between LLZO and copper. We suggest that cracks of this type may be prevented when the current collectors are mechanically compliant and thin compared to the electrolyte layer. Empty cracks in solid electrolytes do not directly lead to electrical short circuits.^33^ All cells could be operated beyond the point where cracks have formed. Once cracks are present in the LLZO and dissolution continues, we found that voids preferably nucleate directly on top of the crack (Figure 9c). Aspects that can enable void nucleation and growth at these sites are (i) the freshly exposed lithium surface may be already considered as a nucleated void at the interface. The lithium surface collects vacancies and then grows along the lithium-LLZO interface, and (ii) the plastic deformation of lithium metal in the proximity of the crack can locally weaken the interface or even lead to delamination. The observation of void nucleation highlights the importance of the interfacial strength of the lithium-LLZO interface. Void growth beyond a critical size requires sufficient levels of stress that depend on the interface energy and surface energies, γ_S,_ and γ_LLZO,_ of a void. Void growth is driven by tensile stress at the interface, and for a given level of stress, void growth is facilitated at sites with reduced interfacial energy.

## Conclusions

Our experimental observations demonstrate the importance of mechanics for ASSBs containing alkali metal anodes with their low melting temperatures. The electrochemistry ends directly at the interface, and mechanical stresses take over as the driving force for lithium metal motion within the electrode: The metal in the electrode is at constant potential, and classical electrochemical mechanisms do not drive metal atoms. Instead, mechanisms known from creep of metals at high temperature move lithium metal to and from the electrochemically active interface. The driving forces are gradients of the vacancy concentration that are equivalent to gradients in internal hydrostatic stress. The observations here are made without stack pressure. It can be expected that external stress not only improves interfacial resistance but also alters some of the effects reported here. Furthermore, the results highlight the strong effect of the microstructure of lithium on the growth and shrinkage of voids in lithium metal in contact with solid electrolytes. A fully reversible evolution of the shape of the electrode cannot be expected when complex microstructures are present. Nevertheless, our observations prove that in principle, voids can grow and shrink without massive shape changes. The observations indicate that high degrees of reversibility can be achieved in single-crystal regions at low rates. Our cells tolerate cracks in the thin electrolyte so that further cell operation under the presence of cracks becomes possible. ASSBs based on Li-LLZO can be reversible, but they are strongly affected by the microstructure, electrochemical interfaces, and passivation layer. Our results indicate that reliable cells might become feasible by tailoring the microstructure of lithium and optimizing the electrochemical interface.

## Supplementary Material

















## Data Availability

The full data set of the experiments presented here is available in the repository KITopen.^40^

## Notes

Overview sample preparation, electrochemical data, additional observations (PDF)
VideoS1_Figure 3 (AVI)
VideoS2_FigureS4 (AVI)
VideoS3_Figure 4 (MP4)
VideoS4_Figure 5 (MP4)
VideoS5_Figure 6 (MP4)
VideoS6_Figure 8_part1 (MP4)
VideoS7_Figure 8_part2 (MP4)
