# Supplementary material for: Formation, Growth, and Shrinkage of Voids in Lithium Metal in Contact With an LLZO Electrolyte
Source: ACS Appl Mater Interfaces. 2025 Oct 5;17(41):56980–90. doi: 10.1021/acsami.5c09594 (PMC12532089; doi:10.1021/acsami.5c09594)
Supplement: Supplementary file 1 [file am5c09594_si_001.pdf]

Supporting information

Formation, Growth, and Shrinkage of Voids in Lithium Metal in contact With an LLZO Electrolyte

Sabrina Lang[1], Lukas Hennerici[2,3], Dominik Kramer[1,], Diana Avadanii[1], Stefan Mück[1,4], Mario Linz[2,3], Jaroslaw Kita[2,3], Ralf Moos[2,3], Reiner Mönig[1,*]*

[1] Institute for Applied Materials, Karlsruhe Institute for Technology, Hermann-von-Helmholtz-Platz 1, 76344 Eggenstein-Leopoldshafen, Germany

[2] Department for Functional Materials, University of Bayreuth, Universitätsstraße 30, 95447 Bayreuth, Germany

[3] Bavarian Center for Battery Technology (BayBatt), University of Bayreuth, Weiherstraße 26, 95448 Bayreuth, Germany

[4] Karlsruhe Nano Micro Facility (KNMFi), Germany

Corresponding Authors: Reiner Mönig (reiner.moenig@kit.edu),
Dominik Kramer (Dominik.kramer@kit.edu)

Table S1. Overview sample preparation.

Experiment	Sample prep.	Coarse Grained/Fine Grained	Applied current	Corresponding Figures
1	80 °C overnight – 230 °C 5 min	CG	± 37 nA	2a-d, 9
2	80 °C overnight – 230 °C 5 min	CG	± 37 nA	3, S4
3	100 °C overnight	FG	- 33 nA	1b, 4
4	Pressed solidified droplet at RT	FG	± 3 nA	5, 7, S8
5	100 °C overnight	FG	- 6 nA	6
6	100 °C overnight	FG	± 10 nA	2e,f , 8
7	100 °C overnight	FG	- 8 nA	S2
8	100 °C overnight	FG	± 5 nA	S10

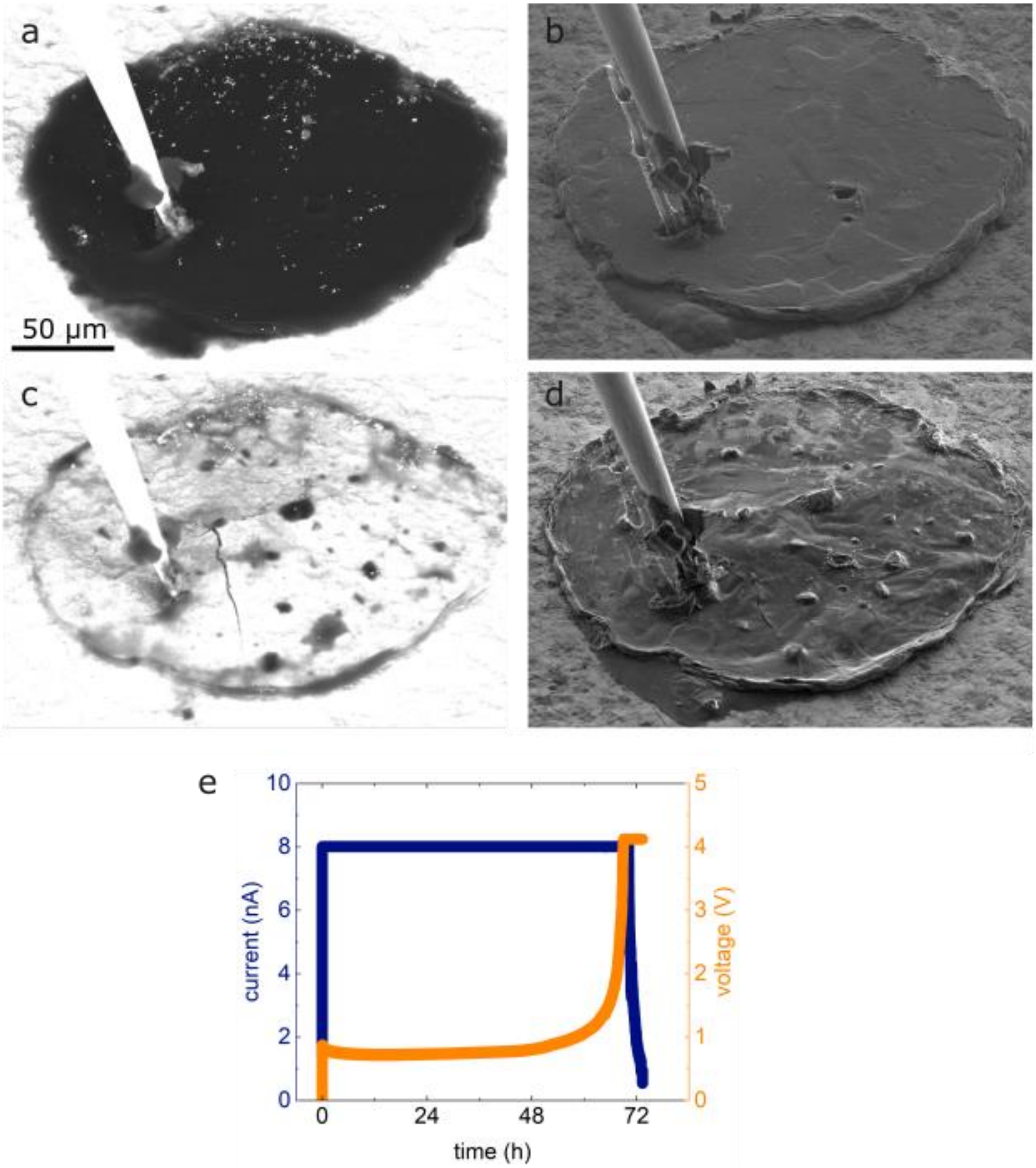

Figure S2. BSE images (18 kV) (a) Initial. c) after dissolution. SE images (3 kV) (b) Initial. (d) After dissolution. (e) Electrochemical data.

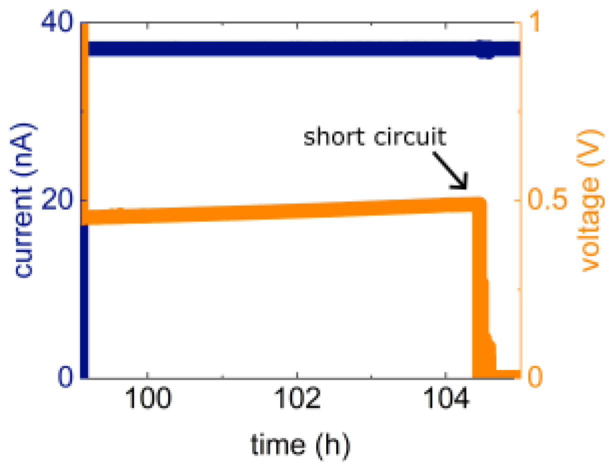

Figure S3. Electrochemical data corresponding to Figure 1 and Figure 9.

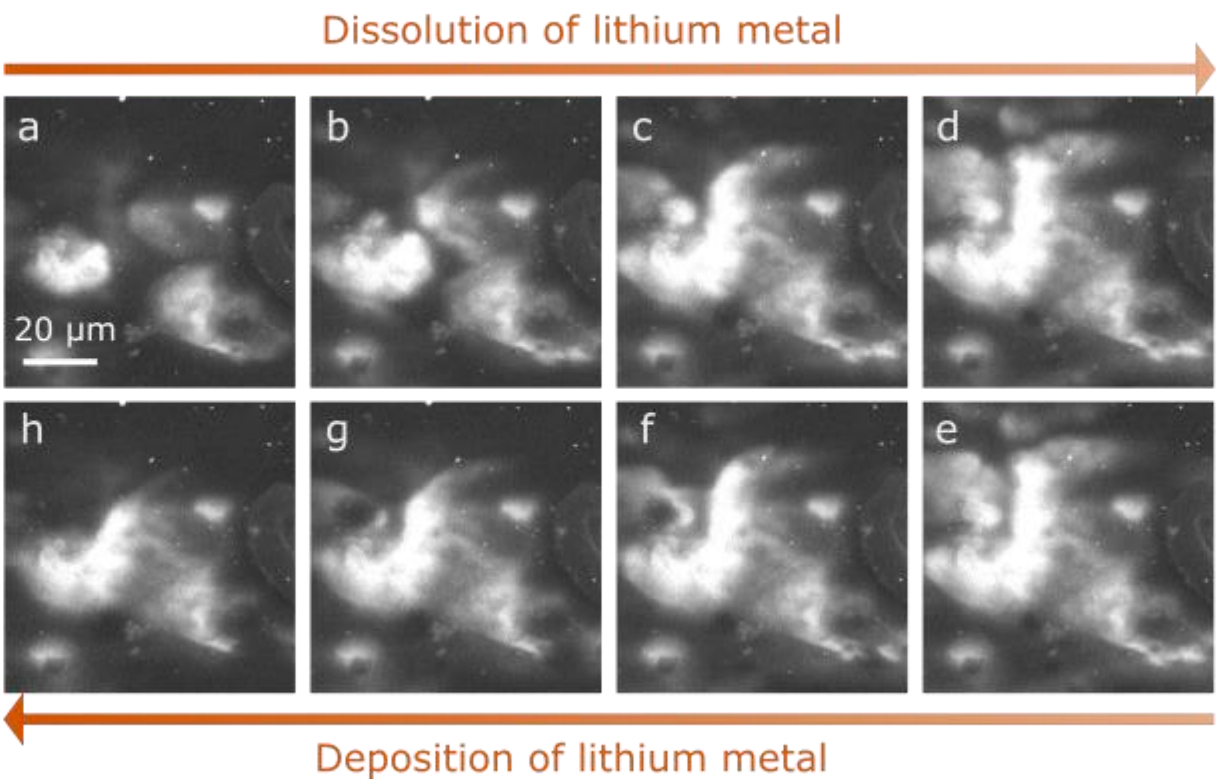

Figure S4. Void growth and replenishment with equal dissolution and redeposition times (a)-(d) BSE images (18 kV) of growth and coalescence of three pores (e)-(h) Replenishment of larger void. Electrochemical data are shown in Figure 3a. Video V2 shows the full data set of the experiment including the initial state of the lithium electrode.

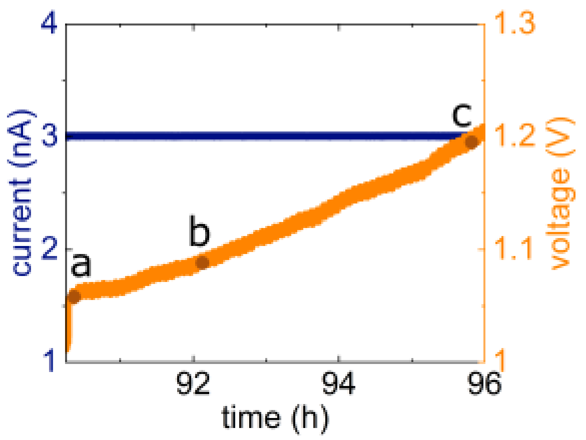

Figure S5. Electrochemical data corresponding to Figure 5.

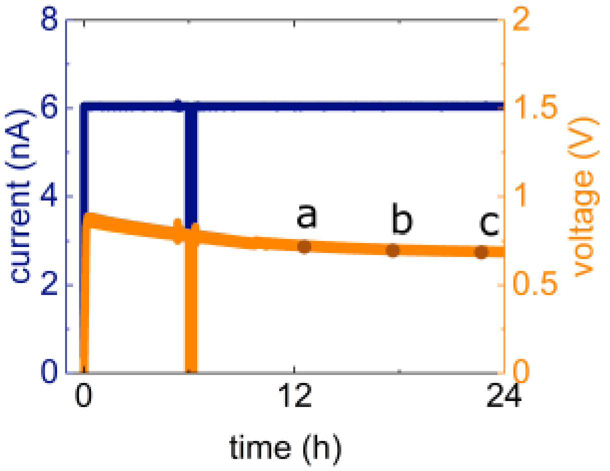

Figure S6. Electrochemical data corresponding to Figure 6.

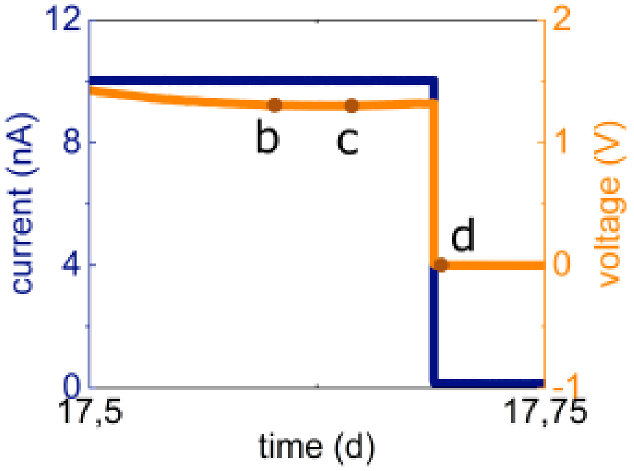

Figure S7. Electrochemical data corresponding to Figure 8.

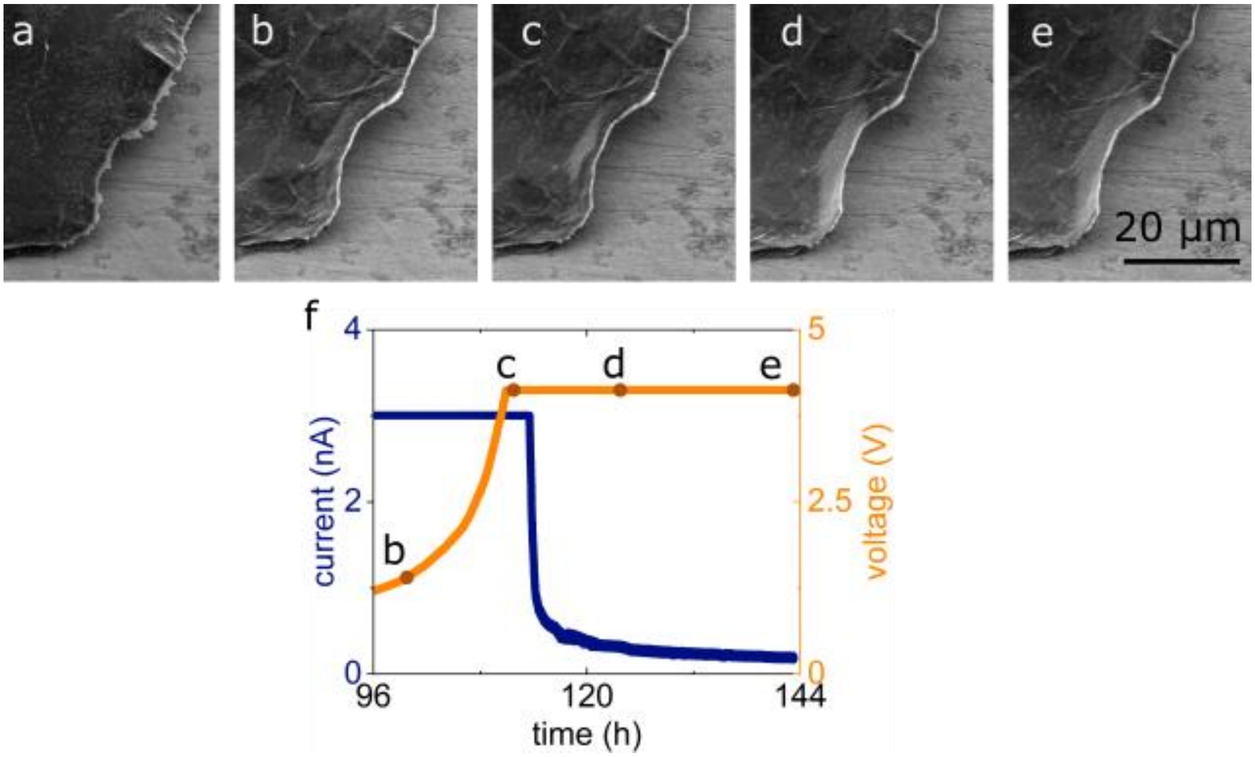

Figure S8. SE images (3 kV) of a pore growing into a detached edge of a lithium metal electrode (a) Initial state. (b)-(e) Growth of the pore. (f) Electrochemical data.

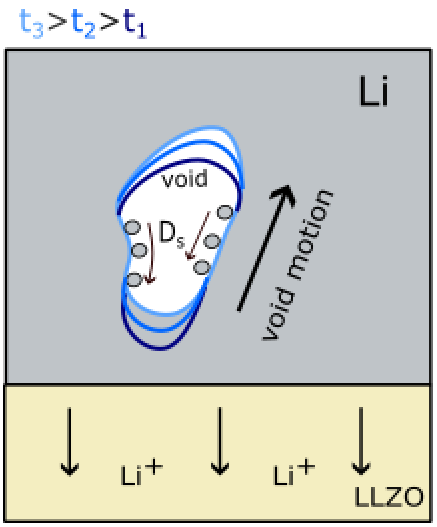

Figure S9. Transport of lithium atoms along the void walls within a lithium metal electrode leads to motion of the void. This transport mechanism could enable fast lithium transport through the bulk. The void moves along the gradient of the stress field in the metal.

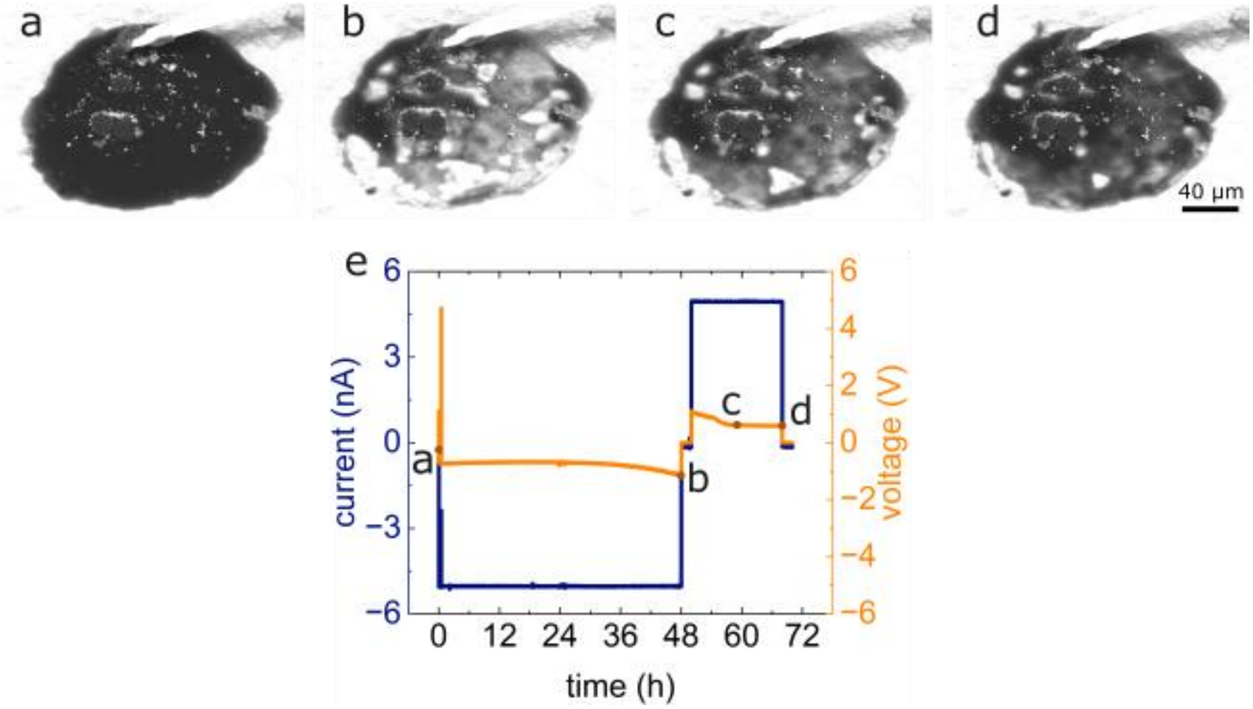

Figure S10. (a-d) Dissolution and redeposition of lithium. (e) Electrochemical data. The passivation layer helps to maintain the electrode shape: In (b) lithium is fully removed under the passivation layer at several regions and reappears later in (c) and (d).
